# Rapid evolution of tolerance to toxic *Microcystis* in two cladoceran grazers

**DOI:** 10.1038/srep25319

**Published:** 2016-04-28

**Authors:** Xiaodong Jiang, Han Gao, Lihua Zhang, Huishuang Liang, Xiao Zhu

**Affiliations:** 1 School of Life Sciences, East China Normal University, Shanghai, 200241, People’s Republic of China

## Abstract

Evolutionary adaptation could assist organisms to cope with environmental changes, yet few experimental systems allow us to directly track evolutionary trajectory. Using experimental evolution, evolutionary tolerance to *Microcystis aeruginosa* was investigated in two cladocerans (*Daphnia pulex* and *Simocephalus vetulus*) to test the hypothesis that cladoceran grazers rapidly adapt to toxic cyanobacteria. After exposure for either three or six months, both grazers evolved a higher tolerance. The intrinsic rate of population increases in *S. vetulus* feeding on cyanobacteria was negatively correlated with that on green algae, which suggests that evolutionary adaptation in tolerance would carry a cost in the absence of cyanobacteria. However, the cyanobacterial selection resulted in a general increase in *D. pulex* when fed both cyanobacteria and green algae. Following a three-month relaxation of selection, *S. vetulus* in the selection line exhibited reverse evolution back to their original state when their diets were switched back to pure green algae. The present experimental evolution, both forwards and reverse, not only demonstrates the evolutionary responses of cladoceran grazers to toxic cyanobacterial cells in the laboratory, but also indicates that the grazer-cyanobacteria interaction would be an effective system to empirically study rapid evolution to environmental changes.

Environmental changes have shifted the growth/reproduction times of organisms, altered the geographic distributions of populations, changed the composition of communities, and shaped the nature of species interactions^1^,^2^. The magnitude and rate of some environmental changes are likely to override the potential responses of many organisms, leaving them susceptible to decline and extinction^3^. Extinction risk of some organisms can be reduced if they migrate to suitable habitats^4^, develop appreciate phenotypic plasticity^5^, or evolve adaptations to counter stressful conditions^6^.

A wave of recent studies have highlighted that adaptive phenotypic evolution can be rapid across a wide range of taxa^7^. These exciting results imply that adaptive evolution could assist organisms to cope with environmental changes to a greater extent than previously thought^8^. In some unconnected habitats, such as isolated lakes or ponds, adaptive evolution might be the only means for aquatic organisms to avoid extinction^2^. The need for evolutionary changes might be more urgent for aquatic organisms with limited swimming abilities, such as zooplankton, compared with nekton^9^. However, with few exception, adaptive evolution has rarely been considered when predicting biological responses to environmental changes and developing management strategies^8^.

In recent decades, eutrophication and climate changes have caused a rise in toxin-producing cyanobacterial harmful algal blooms (CyanoHABs) in freshwater and coastal systems worldwide^10^. Furthermore, increasing CyanoHABs are likely to accumulate more reactive oxygen species in calm surface waters and may form a positive-feedback loop favoring toxin-producing cyanobacteria^11^. CyanoHABs produce and release a wide variety of toxins or secondary compounds^12^, which can have harmful effects on aquatic grazers, such as impaired feeding, physiological dysfunction, depressed reproduction, and increased mortality^13^. Thus, CyanoHABs are likely to pose new selection pressures on aquatic grazers.

There is some evidence for spatial variation in genetic adaptation to toxic cyanobacteria. Somatic juvenile growth rates of *Daphnia pulicaria* from high-nutrient lakes are less inhibited by toxic cyanobacteria than those from low-nutrient lakes^14^. Tolerance to single-celled *M. aeruginosa* increases with increasing prevalence of CyanoHABs in large-bodied *Daphnia pulex* and small-bodied *Chydorus sphaericus* on a microgeographic level^15^. Spatial studies involving common garden experiments identify the direction of adaptive evolution and the pattern of local adaptation, but may not indicate the speed at which adaptation occurs.

Further, a few studies have explored evolutionary adaptation to toxic cyanobacteria on a temporal scale. By hatching resting eggs deposited over several decades, Hairston *et al*.^16^ showed that the tolerance of *Daphnia galeata* to toxic cyanobacteria increased significantly with eutrophication in Lake Constance. However, the direction of selection may not be very clear in such reconstructed populations because grazer performance integrates the adaptive evolution to toxic cyanobacteria during blooms, the reversal during non-blooms, and other selection forces, such as predation, competition and so on. Experimental evolution is a powerful approach for monitoring evolutionary processes occurring in experimental populations in simulated environments^17^. Gustafsson and Hansson^18^ reported that the ability of *D. magna* to deal with toxic cyanobacteria was improved after a four-week exposure to *Microcystis*. In the present study, we seek to extend this observation by asking the following questions: (1) Can cladoceran grazers rapidly adapt to toxic *Microcystis* within the time span of a typical bloom? (2) If so, is there a cost or trade-off for evolved tolerance? (3) Do they return to ancestral status during a non-bloom period?

## Results

### Rapid evolution of tolerance

**Figure 1:**
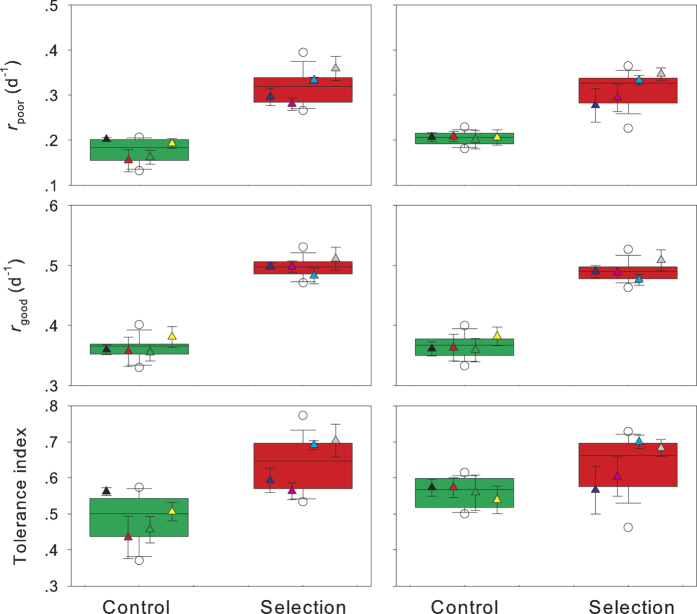
Intrinsic rate of population increase of *Daphnia pulex* feeding on poor (*r*_poor_) or good (*r*_good_) food, and their tolerance index (*r*_poor_/*r*_good_) to cyanobacteria when they were exposed to *Microcystis aeruginosa* for three (left panel) and six (right panel) months. The selection experiment had two lines: the control (green boxplot) and selection (red boxplot), with four replicated tanks in each line. Box plots indicate the median, the 25th and 75th percentiles, with error bars for the 10th and 90th percentiles in grazer performance in an experimental line. Grazer performance in a tank was showed as mean ± s.d. for four replicates of 20 neonates.

Although both the poor (50% *Microcystis aeruginosa* + 50% *Chlorella pyrenoidosa*) and good (100% *C. pyrenoidosa*) food supported positive growth of the large-bodied cladoceran *D. pulex*, the presence of toxic cyanobacteria in diet depressed their intrinsic rates of population increase (Fig. 1). After three months of exposure to toxic cyanobacteria, *D. pulex* in the selection line exhibited significantly higher *r*_poor_, *r*_good_, and tolerance index than their conspecifics in the control line (Fig. 1, two-way nest ANOVA, *P* < 0.05 for all). A similar pattern was observed when *D. pulex* were exposed to *M. aeruginosa* for six months (Fig. 1).

**Figure 2:**
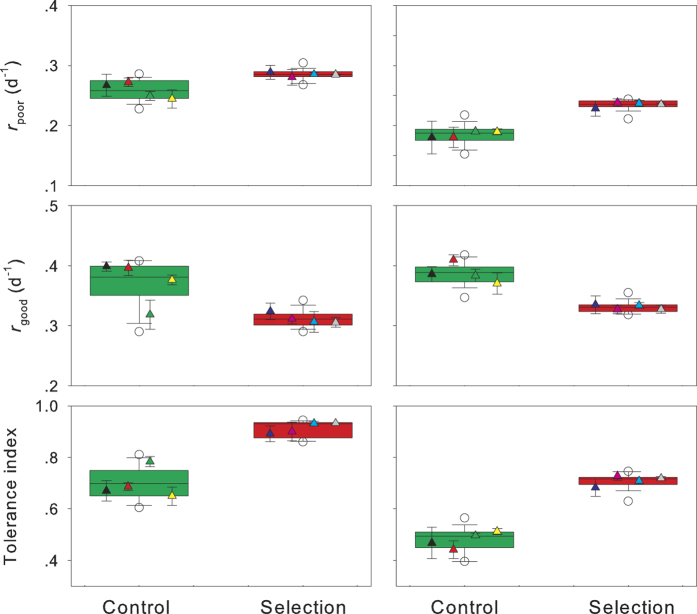
Intrinsic rate of population increase of *Simocephalus vetulus* feeding on poor (*r*_poor_) or good (*r*_good_) food, and their tolerance index (*r*_poor_/*r*_good_) to cyanobacteria when they were exposed to *Microcystis aeruginosa* for three (left panel) and six (right panel) months. Experimental design and figure items as Fig. 1.

The medium-bodied cladoceran *S. vetulus* fed poor food also exhibited the lower intrinsic rates of population increase than the same set of populations fed good food (Fig. 2). After a three-month selection period for tolerance to toxic cyanobacteria, *S. vetulus* in the selection line had significantly higher *r*_poor_ and tolerance index (two-way nest ANOVA, *P* < 0.01 for both), but lower *r*_good_ (*P* = 0.02), than those in the control line (Fig. 2). When the selection time was extended from three to six months, *S. vetulus* exhibited a similar difference between the selection and the control lines (Fig. 2).

### Cost and reversibility of evolved tolerance

**Figure 3:**
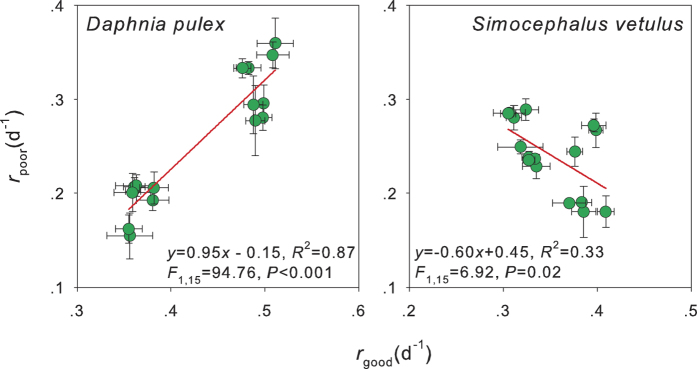
Intrinsic rate of population increases of two grazers when fed poor food versus that of individuals fed good food. Each data point (mean ± s.d.) represents the grazer performance in a tank either before or after the experimental evolution.

**Figure 4:**
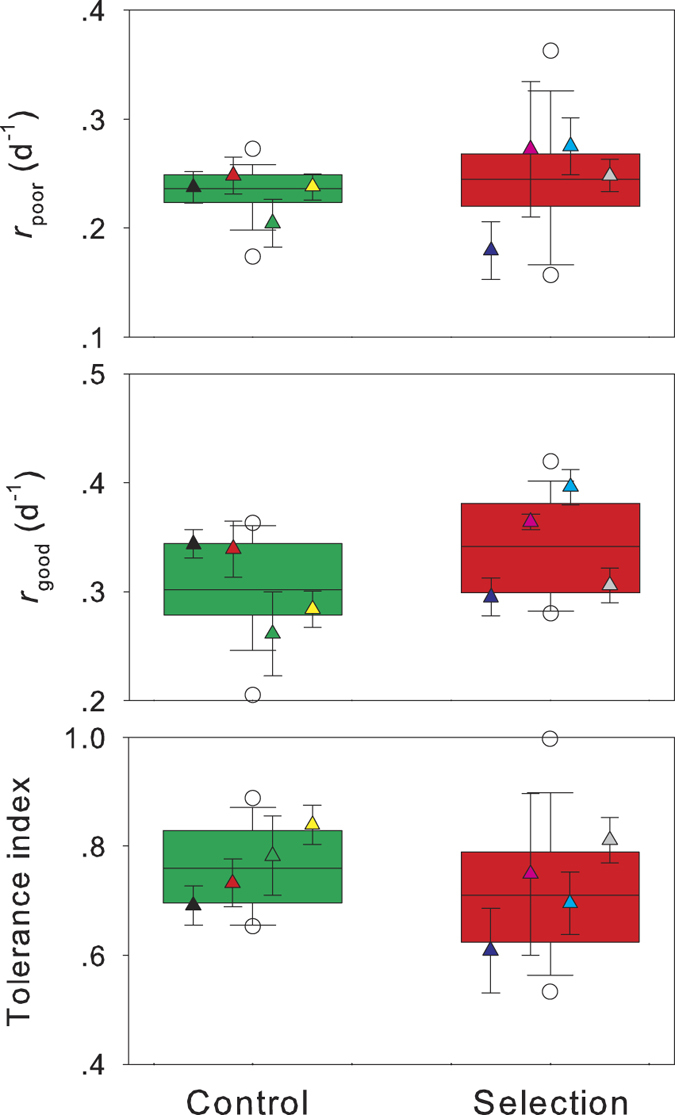
Intrinsic rate of population increase of *Simocephalus vetulus* feeding on poor (*r*_poor_) or good (*r*_good_) food, and their tolerance index (*r*_poor_/*r*_good_) to cyanobacteria when grazers obtained from the selection experiment (Fig. 2) were exposed to *Chlorella pyrenoidosa* for three months. Figure items as Fig. 1.

In the evolution of tolerance to toxic cyanobacteria, *r*_poor_ was positively correlated with *r*_good_ in *D. pulex* (Fig. 3). In contrast, *r*_poor_ was negatively correlated with *r*_good_ in *S. vetulus* (Fig. 3). The exposure to *M. aeruginosa* was halted when *S. vetulus* evolved higher tolerance in the selection line. After feeding on pure *C. pyrenoidosa* for three months, the differences in *r*_poor_, *r*_good_, and tolerance index were not significant between the selection and control lines (Fig. 4, two-way nest ANOVA, *P* > 0.05 for all).

## Discussion

The present results of experimental evolution are consistent with previous reports on the adaptive evolution of tolerance to toxic cyanobacteria from the viewpoints of local adaptation^14^,^15^ and paleolimnological records^16^. Furthermore, our results clearly demonstrated that this evolutionary process occurred within a short time span may be a general response to toxic cyanobacteria for cladoceran grazers via extending test animal from *Daphnia*^18^ to other species. After three months, both *Daphnia pulex* and *Simocephalus vetulus* developed a higher tolerance to *Microcystis aeruginosa* (30% increase). Most individuals of both grazer species mature and produce offspring on day 5 in our system (25 °C and 400 μg C L^−1^)^19^. Thus, grazer populations are expected to develop approximately 18 generations under the present experimental conditions. Such a rapid evolution of tolerance in grazers may occur during CyanoHABs in nature^16^. Selection intensity of toxic cyanobacteria in nature would be more intense due to their higher concentrations. Typical cell densities of cyanobacteria usually range from 10^5^ to 10^6^ cells mL^−1^ during blooms^20^, which greatly exceeds the level in the present selection line (3.4 × 10^4^ cells mL^−1^). The strain of *M. aeruginosa* used in the present study produces microcystins with 24 fg cell^−1^, which translated to a microcystin level of 0.7 μg L^−1^ in the selection line^15^. Such a microcystin level is usually lower than field values during CyanoHABs^21^. However, *Microcystis* usually form colonies to reduce feeding activities by zooplankton grazers^13^. High cell densities and long persistence of cyanobacterial blooms may not certainly imply high selective pressure on zooplankton grazers. On the other hand, field populations of aquatic grazers usually harbor large genetic variation for tolerance to toxic cyanobacteria, which serves as the basis for rapid evolution. Large clonal variation in tolerance to toxic *M. aeruginosa* has been observed in both *D. pulex*^19^ and *S. vetulus*^22^, even within a population. In addition, field populations of grazers are subject to strong gene flow, which may homogenize adaptive evolution^9^. Given the variations in selective force and genetic structure, one must take care in extrapolating rapid evolution in laboratory studies to field populations. Further studies that use field populations of grazers and cyanobacteria in laboratory experiments or directly track evolutionary trajectory in nature are necessary and would provide stronger evidence for rapid evolution of zooplankton tolerance to CyanoHABs.

The ultimate cause of rapid evolution of tolerance to toxic cyanobacteria in two grazers is not clear. Evolution with environmental changes in organisms usually depends on pre-existing genetic variation, de novo mutation for new alleles, or immigration of adaptive individuals^23^. The present experimental design did not consider grazer dispersal, which clearly ruled out the possibility of immigration of adaptive individuals. De novo mutations for new alleles is also not likely to account for the rapid evolution of tolerance in both grazers, not only because the experimental time (three months or 18 generations) is usually too short for mutation, but also because the increased tolerance occurred in all four independent tanks in the selection line rather than in a random one. Rapid evolution of tolerance in both grazers within three months may stem from selection on pre-existing genetic variation. Toxic cyanobacteria may select tolerant genotypes and change the gene frequency of grazers, which would enhance grazer tolerance to toxic cyanobacteria at the population level. Interestingly, we did not observe more divergence between the selection and control lines when the selection experiment was extended from three months to six months. The moderate divergence in tolerance may reflect the limited genetic variation for tolerance and relatively weak selection pressure in our experimental systems since evolutionary rate is expected to be related to the amount of genetic variation in populations and the strength of selection force^24^. Each experimental tank received an initial inoculum of approximately 200 individuals. Furthermore, these individuals were exposed to a relative low level of toxic cyanobacteria (3.4 × 10^4^ cells mL^−1^). Compared with wild populations, the limited population size and the relatively low concentration of cyanobacteria might serve as constraints on the evolution of greater tolerance. The genetic analyses on experimental populations exposed to toxic cyanobacteria may provide more rigorous understandings for processes and mechanisms of increased tolerance in cladoceran grazers. The further studies on zooplankton abundance, fitness, genetic structure, and their correlations with the development of cyanobacterial blooms would decipher zooplankton responses into physiological or evolutionary adaptations, determine the magnitude and rate of adaptive evolution to increasing cyanobacteria in nature, and provide new insights on zooplankton population dynamics and their grazing control on cyanobacterial blooms. Regardless of the precise mechanisms, increased tolerance to toxic cyanobacteria via rapid evolution may buffer mortality risk and enhance grazing pressure in grazers, and consequently affect the dynamics of CyanoHABs.

All adaptations that enhance fitness essentially carry costs to organisms because additional resources are invested to activate necessary gene expression and biochemical processes^5^. These costs, however, have not been well documented. Better estimations of benefits and costs of evolutionary tolerance in terms of individual performance and especially fitness will be critical for any serious attempt to predict evolutionary trajectory of this trait^6^. The genotypes of a marine copepod with greater tolerance to a toxic dinoflagellate carry a substantial reproductive cost when feeding on nutritious algae, which would prevent the fixation of tolerant alleles in natural populations^25^. Rapid evolution of tolerance enhanced the intrinsic rates of population increase of *S. vetulus* in the presence of cyanobacteria, but depressed them in the absence of cyanobacteria, when compared with individuals in the control line. This fitness cost in the absence of cyanobacteria would serve as a constraint on the evolution of tolerance. The evolved tolerance to toxic *M. aeruginosa* in *S. vetulus* was completely lost following a three-month relaxation of selection.

Interestingly, the intrinsic rate of population increases of *D. pulex* on poor food was positively correlated with that on good food. Exposure to toxic *M. aeruginosa* resulted in a general increase in grazer performance on both poor and good food, rather than a trade-off between two conditions. This result is consistent with the previous finding that the juvenile growth rate of *D. galeata* feeding on toxic cyanobacteria is positively correlated with that on nutritious green algae^26^. Aquatic grazers with high growth rates usually have large adult body size, making them more vulnerable to predation^27^. Because actual growth is depressed in all genotypes during CyanoHABs, the combined selection by toxic cyanobacteria and fish predation in turn would favor the dominance of fast-growing genotypes of daphnids^26^. Compared with the large bodied *D. pulex* (2.17 ± 0.12 mm), the medium-bodied *S. vetulus* (1.33 ± 0.03 mm) is less vulnerable to predation, either by a fish predator *Gambusia affinis* (51.0 ± 6.6 vs. 26.9 ± 4.6 prey ind.^−1^ h^−1^, authors’ unpublished data) or by a coelenterate predator *Hydra oligactis*^28^, due to its relatively small size and less movement. The difference in vulnerability to predation in these two grazers may make them suffer different selection pressures in nature and contribute to their differences in the evolutionary trajectory of life history traits. Most studies on zooplankton responses to cyanobacteria have focused on a few *Daphnia* species, ignoring the high diversity in zooplankton^13^. The similar, but not same, evolutionary responses in two cladocerans suggest that interspecific variation should be incorporated when predicting adaptive evolution to toxic cyanobacteria in grazers.

Aquatic grazers are faced with ever-changing CyanoHABs. A particularly interesting question is whether evolved tolerance in grazers would be reversed when CyanoHABs disappear. Reverse evolution is defined as the reacquisition by derived populations of the same character states as those of ancestor populations, which is neither inevitable nor impossible^29^. *S. vetulus* in the selection line, which evolved high tolerance after six months of exposure to *M. aeruginosa*, exhibited reverse evolution back to their original state when their diets were switched back to pure green algae. Similar results have been obtained in experimental studies of evolutionary tolerance to toxic dinoflagellate in a marine copepod^30^. Some ecological variables, such as water temperature, predators, and competitors, usually vary when CyanoHABs disappear with season^12^. Aquatic grazers may face not only the relaxation of cyanobacterial selection, but also emerging selective forces. Testing the reversal evolution under field conditions is expected to bring more insights into evolutionary processes of aquatic grazers.

Phenotypic variance in tolerance did not vary significantly when selection was extended from three to six months, which suggests that there may be still sufficient genetic variation for recovery. Reversion to low tolerance in *S. vetulus* implies that the tolerant genotype may carry a fitness cost in the absence of cyanobacteria, i.e., the lower intrinsic rates of population increase in the absence of *M. aeruginosa*. The experimental evolution, forwards or reverse, in this study suggests that the harmful effects of toxic cyanobacteria cannot always be eliminated, although natural selection can lessen these consequences by promoting adaptation. Additionally, the present study shows that grazer-cyanobacteria interactions could be a good system for empirical study on evolutionary adaptation to environmental changes over short time spans.

## Methods

### Collection and culture of organisms

The microcystin-producing cyanobacterium *Microcystis aeruginosa* (FACHB-905) and the nutritious green alga *Chlorella pyrenoidosa* (FACHB−15) were provided by the Freshwater Algae Culture Collection at the Institute of Hydrobiology, the Chinese Academy of Sciences. They were grown at 25 °C under fluorescent lights at 50 μmol m^−2^ s^−1^ on a 12:12 h light:dark cycle. Phytoplankton cultures were incubated in exponential growth phases with frequent transfers into BG-11 medium. *M. aeruginosa* grow as single or paired cells under the laboratory conditions, which can minimize their mechanical interferences with zooplankton^13^. The carbon contents of *M. aeruginosa* and *C. pyrenoidosa* are 3.81 and 2.59 pg cell^−1^, respectively, estimated by their cell volumes^15^. Phytoplankton suspension for grazers were prepared by measuring the light absorption of a culture in a spectotrophotometer and diluting it with 0.45-μm-filtered tap water with a pre-established linear regression between light absorption and carbon content.

Using a 200-μm mesh net, zooplankton samples were collected from Shangyi Pond (31°01′38″N, 121°26′52″W), Shanghai, China. The low value of microcystins in the sediments (0.30 ± 0.08 μg g^−1^) indicates that cyanobacteria are present, but rarely form blooms in the sampling site^15^. Approximately 1600 adults of either *Daphnia pulex* or *Simocephalus vetulus* were isolated from zooplankton samples.

### Selection experiments

Grazers were randomly split into two lines, the selection line and the control line. For each line, there were four independent tanks filled with 5 L of 0.45-μm-filtered tap water. Individuals in the control lines were fed pure *C. pyrenoidosa* daily during the entire experiment, while those in the selection line were offered a mixed diet of 75% *C. pyrenoidosa* and 25% *M. aeruginosa*, at a food concentration of 400 μg C L^−1^. The relative low cell density of *M. aeruginosa* (3.4 × 10^4^ cells mL^−1^) ensured the population persistence of grazers in the selection experiments, and represented the initial phase of a bloom in which single cells of cyanobacteria are common^12^. They were raised at 25 °C under dim light and half of water in tanks were renewed every four days. Their population density was 40 ind. L^−1^ (200 individual per tank) at the beginning of experiments and was kept under a level of 100 ind. L^−1^ to avoid crowding by randomly discarding individuals when necessary.

Given that CyanoHABs usually persist for several months in the field^20^, the selection experiments ran for either three or six months. At each time point, life table experiments were carried out to measure the intrinsic rate of population increase of grazers.

### Life table experiments

Approximately 50 pre-matured individuals were randomly isolated from each tank and were provided with 400 μg C L^−1^ *C. pyrenoidosa*. Their neonates from the third brood within 24 h of birth were collected to initiate a life table experiment with four replicates (20 neonates in a 500-mL beaker). They were provided with either poor food (50% *M. aeruginosa* + 50% *C. pyrenoidosa*) or good food (100% *C. pyrenoidosa*) at 400 μg C L^−1^. This food concentration represents a typical biomass of phytoplankton in eutrophic lakes, especially in summer when CyanoHABs commonly occur^20^. The presence of 50% *M. aeruginosa* in the diet (6.8 × 10^4^ cells mL^−1^) mimics the phytoplankton community in initial phase of CyanoHABs^20^, and seriously depresses the growth and reproduction of *D. pulex*^15^ and *S. vetulus*^22^. Food selection may be slight because both grazers are generalist feeders^31^,^32^ and both prey organisms have similar shape and diameter size (3.71 ± 0.42 μm for *M. aeruginosa* and 3.28 ± 0.52 μm for *C. pyrenoidosa*) under the present conditions. The number of survivors and their newborn neonates were counted and half of algal suspension was renewed daily. Life table experiments were conducted for ten days at 25 °C because the extension of experimental period contributes a little to the estimation for intrinsic rate of population increase in the present system (approximately 3%, authors’ unpublished data).

### Relaxation experiments

When evolved tolerance in *S. vetulus* was evident in the selection line after the six-month exposure to *M. aeruginosa*, their diet was switched to 100% *C. pyrenoidosa*. After three months, grazer performance on two types of food was measured using life table experiments, to test whether evolved tolerance is reversible.

### Data analysis

Intrinsic rate of population increase (*r*) was calculated iteratively by the Euler-Lotka equation:


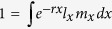


where *l*_*x*_ is the survival proportion at day *x*, and *m*_*x*_ is the number of newborn neonates produced at day *x*. Intrinsic rate of population increase integrates both survival and reproduction and is a more reliable proxy for population fitness. A tolerance index (*T*) to toxic cyanobacteria was calculated as the relative change in intrinsic rate of population increase on poor food (*r*_poor_) compared to that on good food (*r*_good_): *T* = *r*_poor_/*r*_good_^16^. A higher value of this index indicates greater tolerance to toxic cyanobacteria in grazers^15^.

Grazer performance between the treatment lines was compared using two-way nested ANOVA. The tank was nested within the treatment line and was treated as a random effect. Data homogeneity and normality were confirmed by Leven’s test and Kolmogorov-Smirnov test prior to ANOVA. A linear regression was used to explore the relationship between *r*_poor_ and *r*_good_. All statistical analyses were performed using the Statistical Product and Service Solution (SPSS) 16.0 statistical package.

## Additional Information

**How to cite this article**: Jiang, X. *et al*. Rapid evolution of tolerance to toxic *Microcystis* in two cladoceran grazers. *Sci. Rep*. **6**, 25319; doi: 10.1038/srep25319 (2016).
